# Ocular morphologic traits in the American Cocker Spaniel may confer primary angle closure glaucoma susceptibility

**DOI:** 10.1038/s41598-022-23238-1

**Published:** 2022-11-08

**Authors:** Sangwan Park, M. Isabel Casanova, Danika L. Bannasch, Nicole L. Daley, Soohyun Kim, John Kuchtey, Filipe Espinheira Gomes, Brian C. Leonard, Kathryn L. Good, Bianca da C. Martins, Christopher J. Murphy, Sara M. Thomasy

**Affiliations:** 1 Department of Surgical and Radiological Sciences, School of Veterinary Medicine, University of California-Davis, Davis, CA 95616 USA; 2 Department of Population Health and Reproduction, University of California-Davis, Davis, CA 95616 USA; 3 Vanderbilt Eye Institute, Vanderbilt University Medical Center, Nashville, TN 37232 USA; 4 Department of Clinical Sciences, College of Veterinary Medicine, Cornell University Ithaca, New York, 14853 USA; 5 Department of Ophthalmology & Vision Science, School of Medicine, University of California-Davis, Davis, CA 95817 USA; 6 Present Address: Small Animal Specialist Hospital, North Ryde, NSW 2113 Australia

**Keywords:** Translational research, Hereditary eye disease, Glaucoma

## Abstract

Acute primary angle closure glaucoma is a potentially blinding ophthalmic emergency requiring prompt treatment to lower the elevated intraocular pressure in humans and dogs. The PACG in most of canine breeds is epidemiologically similar to humans with older and female patients overrepresented with the condition. The American Cocker Spaniel (ACS) is among the most common breeds observed with PACG development in dogs. This study initially sought to identify genetic risk factors to explain the high prevalence of PACG in ACSs by using a case–control breed-matched genome-wide association study. However, the GWAS failed to identify candidate loci associated with PACG in this breed. This study then assessed intrinsic ocular morphologic traits that may relate to PACG susceptibility in this breed. Normal ACSs without glaucoma have a crowded anterior ocular segment and narrow iridocorneal angle and ciliary cleft, which is consistent with anatomical risk factors identified in humans. The ACSs showed unique features consisting of posterior bowing of iris and longer iridolenticular contact, which mirrors reverse pupillary block and pigment dispersion syndrome in humans. The ACS could hold potential to serve as an animal model of naturally occurring PACG in humans.

## Introduction

Primary angle-closure glaucoma (PACG) poses a significant public health concern and the global population affected with PACG is predicted to increase over 30 million by 2040^1^. Although primary open angle glaucoma (POAG) is the most common form in humans, the likelihood of severe bilateral visual impairment is 3 times higher in PACG versus POAG^2^. Advancing age, female sex, and Asian ethnicity are known predisposing factors in the signalment of patients with PACG^3^. In most canine breeds, PACG shares similar epidemiological characteristics with humans in that females and middle-age to older patients are predisposed^4^. Furthermore, clinical presentations of PACG are also similar between humans and dogs. The PACG diagnosis is often made at the advanced clinical stages showing acute angle closure or chronic visual loss in both humans and dogs^2,4^.

Canine PACG is characterized by eventual ciliary cleft (CC) collapse following progressive narrowing of the CC and iridocorneal angle (ICA)^5,6^. Certain breeds, including the American Cocker Spaniel (ACS), Basset Hound, Chow Chow, Siberian Husky, and others are reported to be predisposed to PACG development with the ACS reported to have the highest prevalence of 5.5% in North America^5,7^. Pectinate ligament dysplasia (PLD), which is a developmental abnormality of the ICA resulting from insufficient remodeling of mesenchymal tissue at the anterior aspect of the CC, is known to be associated with PACG development in dogs^4,5^. Although most canine breeds predisposed to PACG including ACS are affected with PLD^6^, only a small proportion of dogs with PLD will develop PACG in their lifetime^8^. The inheritance of PLD was documented in several breeds including English Springer Spaniel, Flat-Coated Retriever, and Samoyed, and several risk/candidate genes for PACG development such as *COL1A2*, *NEB*, and *SRBD1* were identified in the Basset Hound, Shiba Inu, and Shih-Tzu, respectively, by candidate gene and genome-wide association studies (GWAS)^6^. However, the genetic background of canine PACG has not yet been clarified and it is generally considered to be a complex trait with multiple genetic and environmental factors associated.

Not all dogs with PACG have PLD. But the current consensus on the pathogenesis of canine PACG is that the PLD is the first ‘hit’ which initiates reverse pupillary block in dogs^4^, and a narrow iridocorneal angle (ICA) and ciliary cleft (CC) are prerequisite structural abnormalities for PACG development^6,8^. While PLD alone did not change aqueous outflow capacity and IOP^9,10^, the odds of developing PACG were 20 times higher in dogs with a narrow or closed CC than dogs that did not have this feature, suggesting that the tissues within the trabecular meshwork/CC have a greater impact on aqueous humor outflow capacity than PLD^8^. Most canine studies have focused on ICA narrowing or CC collapse and neglected to evaluate the CC in relation to the surrounding ocular structures, particularly the lens-pupil interface. Indeed, in humans, it is known that ICA narrowing may exist alone, but more often co-exists with pupillary block to cause PACG^11^.

The purpose of this study was two-fold: (1) to identify candidate loci associated with PACG in ACS using a GWAS; (2) to reveal ocular morphologic traits of ACS that may confer the PACG susceptibility in comparison to Beagles which have a similar body size to the ACS but not considered predisposed to PACG.

## Results

### GWAS in PACG-affected and non-affected control ACS

The 28 PACG-affected ACSs included 13 males (3 intact and 10 neutered) and 15 females (4 intact and 11 spayed), whose ages ranged from 4 to 12 years. Twelve ACSs were bilaterally affected, and 16 ACSs were unilaterally affected. Gonioscopic descriptions were collected from 16 affected ACSs, all of which were judged by the examining veterinary ophthalmologist to show segmental goniodysgenesis and/or narrowed iridocorneal angle in at least one eye. Nine affected ACSs were histopathologically confirmed with the diagnosis of goniodysgenesis-related PACG.

The 24 breed-matched controls included 11 neutered males and 13 spayed females, whose ages ranged from 7 to 15 years. Details of ocular findings of 18 control ACSs that were included in both GWAS and imaging will be described in the following imaging section. The 6 control ACSs that were included solely in the GWAS demonstrated normal iridocorneal angle on gonioscopy.

**Figure 1:**
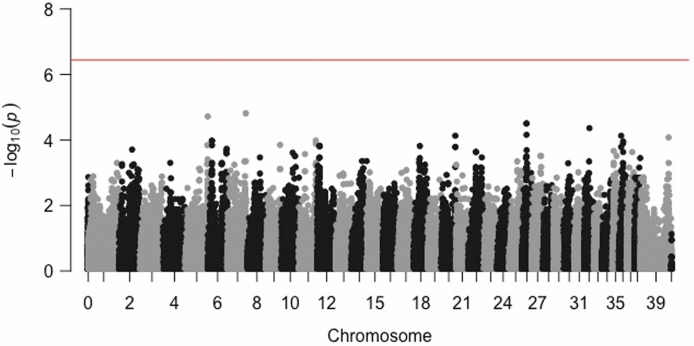
Manhattan plot for GWAS (Cases: 28 PACG-affected; Controls: 24 non-affected) in the ACS. The horizontal red line denotes the threshold for genome-wide statistical association and the black and grey colors alternate each other to delineate each chromosome easily. No statistically significant single nucleotide polymorphisms associated with PACG were identified.

Mean age significantly differed in the non-affected controls versus the PACG-affected ACSs at 10.9 ± 2.3 versus 8.9 ± 1.9 years, respectively (*p* = 0.0015). The case–control GWAS using the 28 PACG-affected cases and 24 non-affected controls in ACS did not show statistically significant single nucleotide polymorphisms (SNP) associations in any regions (Fig. 1). Given that a previous study identified a near-significant association at the CFA10 locus in a case–control GWAS within the ACS breed^12^, we further investigated the genotypes at the CFA10 SNP in the present ACS population—85.7% of affected dogs were homozygous for the A allele compared to 62.5% of controls (Supplementary Table S1).

### Comparative ocular morphological characterization of non-affected ACS and Beagle dogs:

Twenty-four ACSs (13 neutered males and 11 spayed females) and 7 Beagle (2 neutered males, 4 spayed females and 1 sexually intact female) dogs were enrolled in this study. Mean ± SD age was 10.0 ± 2.1 (range 7–15) years for the 24 ACSs and 11.6 ± 2.3 (range 9–16) years for the 7 Beagles; age did not significantly differ between the two breeds (*p* = 0.1057).

All enrolled dogs had normal vision, were systemically healthy and not receiving any medication. On ophthalmic examinations, ocular surface disease including keratoconjunctivitis sicca and corneal vascularization was identified in 3 ACSs. Early cataracts were identified in 12 ACSs (10 had incipient cataracts and 2 had early immature cataracts) and 6 Beagles (5 had incipient cataracts and 1 had early immature cataracts). Iridociliary cysts in the inferior anterior chamber were observed in 1 ACS and 1 Beagle. Segmental goniodysgenesis and/or mildly narrowed iridocorneal angle on gonioscopy was identified subjectively in 4 ACSs and 2 Beagles. Mean ± SD IOP was 14 ± 5 (range 8–19) and 16 ± 3 (range 13–20) mmHg for the ACSs and Beagles, respectively, which was statistically significant (*p* = 0.0452). There was no statistical difference in IOP between males and females for both ACSs and Beagles.

**Figure 2:**
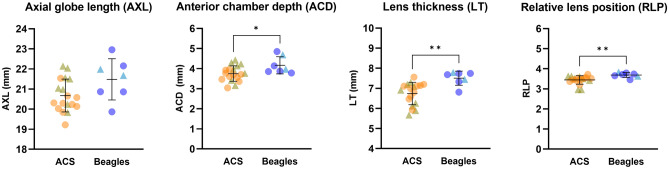
The ACSs have significantly more crowded anterior ocular segment structures. While the overall globe size was not different between the two breeds, the anterior chamber was significantly shallower in ACS versus Beagles. Although the ACS have significantly thinner lenses, the lenses were more anteriorly located in ACS versus Beagles. An unpaired *t*-test was performed (n = 18 ACS and 7 Beagles; * = *p* value < 0.05; ** = *P* value < 0.01). The line and error bars indicate mean values and standard deviations. The circles and triangles indicate females and males, respectively.

A-scan ocular ultrasonography was performed in 18 ACSs (mean age, 10.7 years; 8 neutered males and 10 spayed females) and 7 Beagles. While the axial globe length (AXL) was not statistically different between the two breeds (20.67 ± 0.81 and 21.48 ± 1.02 mm for ACS and Beagle, respectively), there were statistically significant differences in anterior chamber depth (ACD), lens thickness (LT) and relative lens position (RLP) between the two breeds (Fig. 2). The anterior chamber was shallower in ACS (3.75 ± 0.39 mm) versus the Beagle (ACD: 4.17 ± 0.43 mm; *p* = 0.0278). The lens was thinner in ACS (LT: 6.74 ± 0.56 mm) compared to the Beagle (7.50 ± 0.35 mm; *p* = 0.0028) and was more anteriorly located in ACS (RLP: 3.44 ± 0.21 and 3.69 ± 0.12 for ACS and Beagle, respectively; *p* = 0.0039). Within the ACSs, AXL was statistically greater in males versus females (AXL: 21.17 ± 0.82 mm for males and 20.28 ± 0.60 mm for females; *p* = 0.0175) while the other A-scan ocular biometry parameters did not significantly differ between sexes. In Beagles, no statistical differences were identified between males and females for all A-scan ocular biometry parameters.

**Figure 3:**
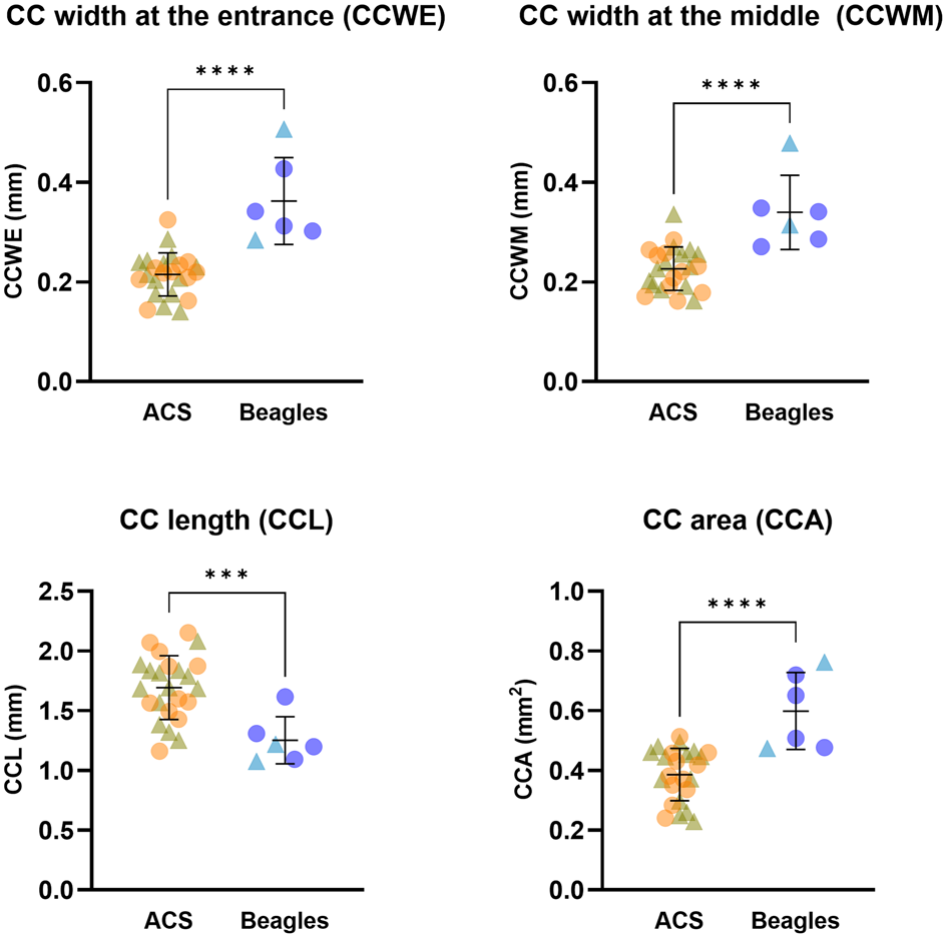
The ciliary cleft (CC) of ACSs was significantly more narrow than that of Beagles. While the CC length (CCL) was significantly longer in ACS, the CC width at the entrance and the middle (CCWE and CCWM) were significantly shorter resulting in a significantly smaller CC area (CCA). An unpaired *t*-test was performed (n = 24 ACS and 6 Beagles; *** = *p* value < 0.001; **** = *p* value < 0.0001). The line and error bars indicate mean values and standard deviations. The circles and triangles indicate females and males, respectively.

Ultrasound biomicroscopy (UBM) was performed in 24 ACS and 7 Beagle dogs. One Beagle dog was excluded from image analysis due to the presence of ciliary cysts in the dorsotemporal posterior chamber OU where the probe was applied in order to avoid their possibility of distorting ciliary cleft and iris configurations^13^. Regarding ciliary cleft-related parameters, most were significantly smaller in ACS versus Beagle dogs (CCWE (ciliary cleft width at the entrance), 0.22 ± 0.04 mm for ACS and 0.36 ± 0.09 mm for Beagle (*p* < 0.0001); CCWM (ciliary cleft width at the middle), 0.23 ± 0.04 mm for ACS and 0.34 ± 0.07 mm for Beagle (*p* < 0.0001); CCA (ciliary cleft area), 0.39 ± 0.09 mm for ACS and 0.75 ± 0.30 mm for Beagle (*p* < 0.0001); Fig. 3). The exception was ciliary cleft length (CCL), which was 1.69 ± 0.27 mm for ACS and 1.25 ± 0.20 mm for Beagle (*p* = 0.0008).

**Figure 4:**
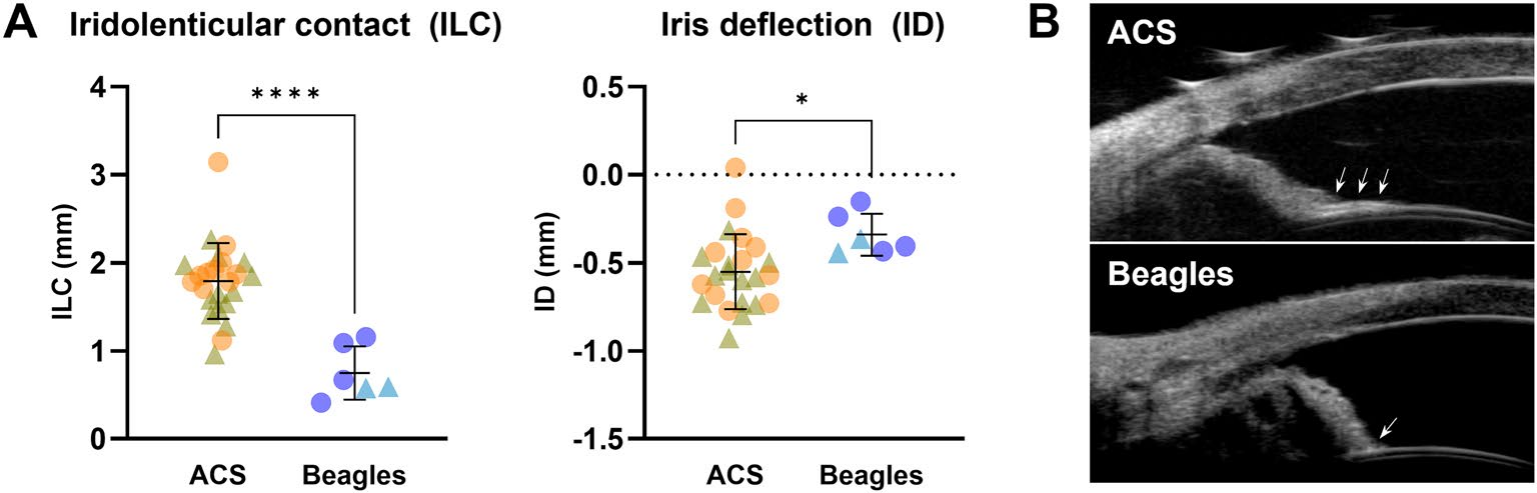
The iris of the ACS was significantly more in contact with the anterior lens capsule and more concave (posteriorly deflected) in comparison to the Beagles. (**A**) The ACS have significantly longer iridolenticular contact (ILC) with a greater degree of posterior iris deflection (ID) versus Beagles. An unpaired *t*-test was performed (n = 24 ACS and 6 Beagles; * = *p* value < 0.05; **** = *p* value < 0.0001). The line and error bars indicate mean values and standard deviations. The circles and triangles indicate females and males, respectively. (**B**) Representative UBM images of ACS and Beagles are shown. Arrows indicate the iris in contact with the anterior lens capsule.

Regarding the iris-related UBM parameters, iris contact with the anterior lens capsule or iridolenticular contact (ILC) was significantly greater in the ACS (1.79 ± 0.43 mm) versus Beagle (0.75 ± 0.30 mm; *p* < 0.0001). In addition, the iris, as measured by iris deflection (ID), in the ACS (− 0.55 ± 0.21 mm) was more concave (posteriorly deflected) compared to the Beagle (− 0.34 ± 0.12 mm; *p* = 0.0271) (Fig. 4). For all UBM parameters, there were no statistical differences between males and females for both ACS and Beagles.

## Discussion

Despite the high prevalence of PACG in ACS, what makes ACS more susceptible to PACG development in comparison to other canine breeds remains unknown. In the present study, GWAS failed to identify candidate loci associated with PACG in ACS. This result in combination with the high incidence of PACG in ACS reiterates that canine PACG could be a multifactorial disease having complex genetic traits^5–7^. While this study included a modest number of samples for a GWAS, the samples were thoroughly phenotyped and the results are consistent with a previous study with a greater number of samples (> 100 samples for cases and controls, respectively) in this breed where no significant association was identified^12^. Given that canine PACG has a female predilection and is considered an adult-onset disease that does not typically develop until 4–10 years of age^7^, matching controls further based on sex and selecting non-affected controls much older than affected cases may be helpful^14^. In the present study, the ocular morphologic traits demonstrated in non-affected ACSs suggest that a large percentage of the ACS breed may be susceptible to PACG development and that this disease is incompletely penetrant.

This study demonstrated that female ACSs tend to have shorter AXLs than males. In addition, the ACS has a more crowded anterior segment morphology with a significantly shallower ACD and thinner but more anteriorly located lens versus the Beagle, although their overall globe size is not statistically different. We highlight that the observed significant differences in ocular biometric results were not age-related as the results obtained from the Beagles of the current study were consistent to a previous study performed in younger Beagles^15^. In humans, shallow ACD, short axial globe length and thereby hyperopic eyes, and thick, anteriorly located lens are known risk factors for acute primary angle closure, all of which contribute to the formation of crowded anterior segment anatomy^3,16,17^. Although the role of the lens has been understudied in veterinary-based ophthalmology, an increase in thickness and a more anterior position of the lens are closely associated with PACG in humans^3,16,18^.

It is interesting to note that the ACS has a thinner lens, which is more anteriorly located in comparison to Beagles. In humans, anterior lens position appears to be a more critical and significant risk factor than lens thickness for angle closure development^19^. More specifically, the lens vault or the magnitude of the lens anterior to the scleral spur plane measured with spectral domain anterior-segment optical coherence tomography (AS-OCT) was a significant predictor of angle closure, and more predictive than traditional lens biometric parameters such as LT and RLP^20–22^. The greater lens vault as well as the anterior position of the lens could lead to anterior displacement of peripheral iris, directly aggravating ICA and CC narrowing^20–22^. It would be worthwhile to investigate the lens vault in various breeds to determine its role in canine PACG development. As dogs do not possess a scleral spur, another landmark is required to represent the plane of angles. In addition, their larger eyes in comparison to humans do not allow capture of the entire anterior ocular segment in a single image with spectral domain AS-OCT. Given these anatomical and technical difficulties, a novel canine-specific parameter needs to be defined to assess how much of the lens is anteriorly protruded relative to other structures.

It is expected that the more anterior location or protrusion of the lens increases pupillary block via increased iridolenticular contact^21^. In the present study, contact between the posterior iris surface and the anterior lens capsule was significantly greater in ACS. This increased ILC reinforces the one-way valve at the pupillary margin that traps more aqueous in the anterior chamber, inducing the concave sigmoidal-shaped iris as quantified by the more negative ID parameter in ACS versus Beagle^23^. The co-existence of the concave iris and angle narrowing/closure is a unique finding in dogs. In humans, PACG is associated with relative pupillary block characterized by anterior bowing (convex) of iris causing narrowing of the angle^11,24^. The reverse pupillary block characterized by posterior bowing (concavity) of the iris is observed in pigment dispersion syndrome and pigmentary glaucoma, which is considered secondary open-angle glaucoma caused by pigment accumulation on trabecular meshwork with increased outflow resistance^25^. Iris concavity was observed in 38–54% of humans affected with pigment dispersion syndrome^26,27^, 45% with latent pigmentary glaucoma and 86% with active pigmentary glaucoma^27^. However, reverse pupillary block is not pathognomonic for pigment dispersion syndrome and pigmentary glaucoma as blinking, accommodation, and exercise have been reported to cause this phenomenon^25^. Distinguishing between physiological and pathological reverse pupillary block can be challenging but will typically persist in the eyes affected with pigment dispersion syndrome and pigmentary glaucoma while it is temporary in normal human eyes post-exercise^28^. Additionally, as pupillary block is more common in a mid-range pupil and iris area and volume contribute to the shape of iris^29^, assessing pupil size or iris volume would be needed for better understanding of the reverse pupillary block mechanism in dogs.

While IOP was significantly lower in normal ACSs versus Beagles, all dogs had an IOP within the normal range. This may not be clinically relevant, but we can postulate on some possible causes. One possible scenario could be an association with a low-grade subclinical intraocular inflammation that was not detected by routine ophthalmic exams. This hypothesis, in turn, highlights the histopathological findings that lymphoplasmacytic inflammation as well as pigment dispersion were prevalent in canine eyes affected with goniodysgenesis-related glaucoma^30^. Additionally, it corroborates the increased expression of COX-2 in trabecular meshwork, angular aqueous plexus and ciliary body of glaucomatous dog eyes and increased VEGF concentration in aqueous humor of dogs affected with primary glaucoma^31,32^. Indeed, uveoscleral outflow was also more enhanced in humans affected with pigment dispersion syndrome to compensate for the pigment dispersion process compromising the trabecular outflow facility and to maintain a normal IOP^33^. Thus, measurement of uveoscleral outflow facility and sampling of aqueous humor to measure inflammatory mediators in the ACS might help explain the underlying mechanism of lower normal IOPs in this breed in comparison to the Beagle.

Given the small sample size, the finding of lower normal IOP in normotensive ACS needs to be verified with a larger number of animals in a more controlled setting to avoid technical or diurnal IOP variations. In addition, there might be breed-related differences in IOP due to different corneal properties including curvature, thickness and biomechanics that were not assessed in the present study^34,35^. Meanwhile, it is unclear if the inflammatory evidence reported in the literature thus far suggests a primary trigger of glaucoma or is a secondary response to the intermittent episodes of pupil block^30^. Furthermore, it is still unknown whether there is a physical friction between the iris and the lens liberating pigments in vivo despite the observed iris configurations of ACS typical for reverse pupillary block. To elucidate the role of pigment dispersion in PACG development of dogs, the use of more sophisticated measures such as laser flare-cell photometry may be needed to quantify the number of melanin granules or cells floating in the anterior chamber^36^. Moreover, the ACS population enrolled in this study was comprised of ophthalmoscopically healthy, normotensive dogs although the presence of CC narrowing could be categorized as the latent and asymptomatic phase of PACG^4^. Thus, it is unknown how many of these ACSs will develop glaucoma. A longitudinal study assessing the entire anterior ocular segment anatomy including the position and relationship of all anatomical structures would be highly informative to identify the contributing factors for the longer disease-free interval as well as main risk factors for PACG development in the ACS.

In summary, normotensive ACS with normal eyes had a narrowed CC and more crowded anterior ocular segment consisting of a shallow anterior chamber and a more anteriorly located lens in comparison to Beagles. The narrow CC existed concomitantly with increased ILC and posterior bowing of iris, typical features for reverse pupillary block. The resting IOP of the present ACS population was significantly lower than that of Beagles despite the aforementioned unfavorable anatomical features. The nonsignificant results of a case–control GWAS in glaucomatous and normal ACSs could be attributed to the PACG susceptibility of the ACS breed due to their intrinsic ocular morphology. Finally, companion animals have a more heterogenous genetic background compared to laboratory animals and share the same environmental factors with humans^37^. Given that PACG is a complex multifactorial disease with variable expression and low penetrance^2^, the ACS may hold potential to serve as an animal model of naturally occurring PACG. The co-existence of posterior iris bowing with angle narrowing, however, should be kept in mind when utilizing the ACS in translational research.

## Methods

Experimental protocols of this study were approved by the Institutional Animal Care and Use Committee of the University of California-Davis (#19044 and 20700). All the methods were performed in accordance with relevant guidelines and regulations. All the procedures of this study were carried out in compliance with the ARVO Statement for the Use of Animals in Ophthalmic and Vision Research and the ARRIVE guidelines 2.0. Since the PACG diagnosis is made based on IOP values, investigators could not be masked to the case/control group allocation. Prior to study entry, informed and written consent was obtained from owners for all enrolled dogs.

### GWAS in PACG-affected and non-affected control ACS:

The 28 ACS dogs that were diagnosed with PACG in at least one of their eyes by board-certified veterinary ophthalmologists or board-certified veterinary pathologists were included and designated as PACG-affected cases. Clinically, PACG was defined when there was an IOP elevation > 30 mmHg in the absence of antecedent ocular disease and/or goniodysgenesis in at least one eye by gonioscopy^38,39^. When pathology reports of eyes enucleated due to intractable glaucoma were available, samples given a causative diagnosis of goniodysgenesis-related glaucoma were only included for PACG-affected cases. The 24 breed-matched dogs that had normotensive, ophthalmoscopically healthy eyes with no previous history of glaucoma and were at least 7 years of age served as non-affected controls for the genetic study. Venous blood samples were collected, and DNA was extracted accordingly (Gentra Puregene, Qiagen, Hilden, Germany). Twelve DNA samples of ACS cases and 3 DNA samples of ACS controls obtained for a previously published paper^38^ were re-used in this study for GWAS.

Samples with a minimum of 300 ng of DNA at a minimum concentration of 20 ng/μl were genotyped using the Illumina CanineHD 220 K BeadChip array (Illumina, San Diego, CA). CanFam3.1 assembly was used as reference. All samples passed quality control and a case–control association analysis was performed on all SNPs with minor allele frequencies > 5% and missing genotype call rate < 10% by using the software package PLINK 1.9^40^. Bonferroni correction was applied to correct for multiple comparisons using PLINK. All samples and 137,057 variants passed the filters, and the genomic inflation (λ) was 1.10. The population stratification was evaluated with Q-Q plots and multi-dimensional scaling (MDS) plots. The Manhattan plot was built in R (qqman package)^41^.

### Comparative ocular morphological characterization of non-affected ACS and Beagle dogs:

Of the non-affected 24 control ACS dogs, 18 were included in both GWAS and the imaging study. An additional 6 ACS dogs that met the same control criteria were enrolled only for the imaging study. Finally, 7 client-owned Beagle dogs were included. The same inclusion criteria were applied for the Beagle dogs as the non-affected ACS controls as follows: (i) age greater than 7 years and (ii) normotensive, ophthalmoscopically healthy eyes with no previous history of glaucoma. Ophthalmic exams including rebound tonometry, slit lamp biomicroscopy, gonioscopy and indirect ophthalmoscopy were performed. Subsequently, advanced ocular imaging including A-scan ocular biometry and UBM were performed with gentle manual restraint.

A-scan ultrasonography (Sonomed PacScan 300A +, Escalon, Wayne, PA, United States) was used to measure ACD, LT, and AXL in both eyes. The RLP was calculated by adding the ACD to half the LT and then dividing the sum by the AXL, which was then multiplied by 10^18^. Ultrasound velocities were set at 1532 m/s for the anterior chamber and the vitreous body and 1641 m/s for the lens. A 10 MHz A-scan probe was perpendicularly placed over the central cornea with a coupling gel (Goniosoft; OcuSoft Inc., Richmond, TX) following topical anesthesia with proparacaine. Five A-scan recordings were obtained on the manual freeze mode when all the required echoes with sufficient height were present and averaged for each eye.

**Figure 5:**
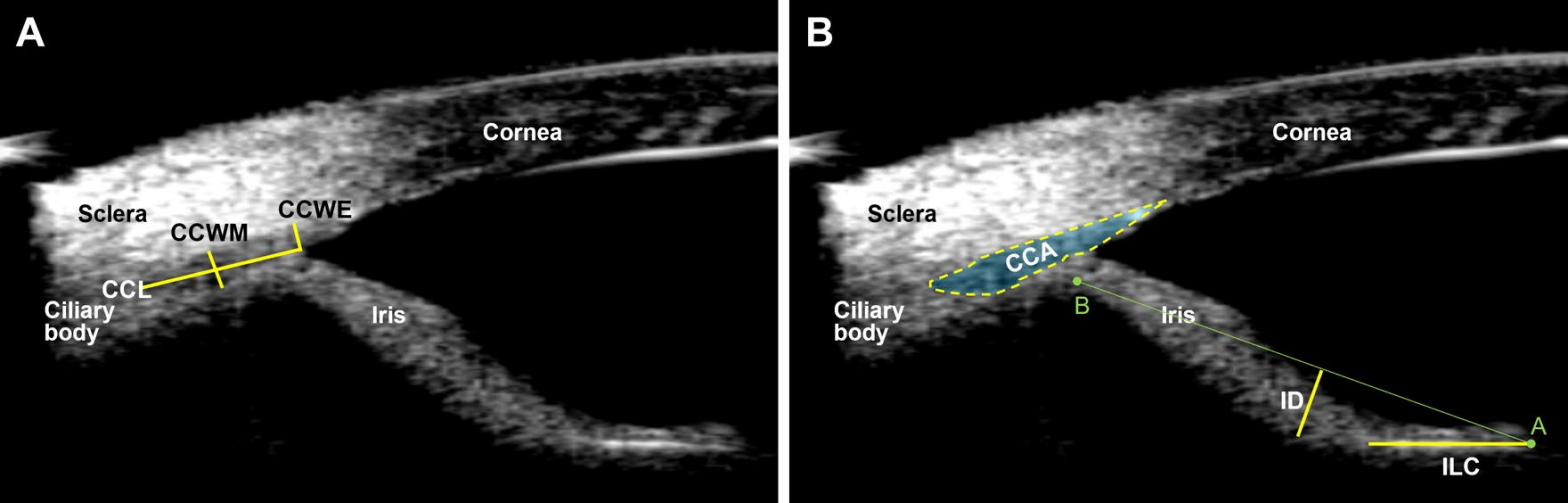
Measurements of UBM parameters (**A**: CCWE, CCWM, and CCL. **B**: CCA, ILC, and ID). CCWE, ciliary cleft width at the entrance; CCWM, ciliary cleft width at the middle; CCL, ciliary cleft length; CCA, ciliary cleft area; ILC, iridolenticular contact; ID, iris deflection.

In order to evaluate the ICA and CC anatomy, UBM scan was performed with a handheld 50-MHz transducer in both eyes. The transducer was placed perpendicular to the dorsotemporal limbus with a coupling gel (Optixcare; Aventix Animal Health, Ontario, Canada) following topical anesthesia. Three UBM images were selected for each eye and the following CC-oriented parameters were assessed for each obtained image: (i) CCWE, defined as the distance on a perpendicular line from the surface of the peripheral iris root to the inner surface of the cornea or sclera; (ii) CCWM, defined as the distance between the inner and outer surface of the internal border of the ciliary cleft at the middle of it, (iii) CCL, defined as the distance between the caudal end of the ciliary cleft and the surface of the peripheral iris root, and (iv) CCA, defined as the area surrounded by the internal border of the ciliary cleft and corneoscleral limbus (Fig. 5).

Furthermore, in order to quantify iris configuration and compare between the two breeds, we measured the following parameters: (i) ILC, defined as the distance between the innermost and outermost point of posterior pigmented epithelium of iris in contact with the anterior lens capsule and (ii) ID, defined as the maximum distance reached by the posterior pigmented epithelium from a line joining the innermost point of the posterior pigmented epithelium of iris in contact with the lens with its outermost point at the iris root (Fig. 5)^26^.

The values for each parameter and each of the 3 images were averaged in each eye.

Descriptive statistics (mean ± SD) were used to summarize demographic and all ocular biometric data. The normality of each set of data was tested using the Shapiro–Wilk test. An unpaired *t*-test was used for comparison of the means of each data set between the two breeds and between the sexes within the breed. A *p* value of < 0.05 was considered statistically significant; GraphPad Prism v9 (GraphPad Software Inc., La Jolla, CA, USA) was used for all analyses.

## Supplementary Information

Supplementary Information.

## Data Availability

All data generated or analyzed during this study are included in this published article [and its supplementary information files].
