# Supplementary material for: Ocular morphologic traits in the American Cocker Spaniel may confer primary angle closure glaucoma susceptibility
Source: Sci Rep. 2022 Nov 8;12:18980. doi: 10.1038/s41598-022-23238-1 (PMC9643544; doi:10.1038/s41598-022-23238-1)
Supplement: Supplementary file 1 — Supplementary Information. [file 41598_2022_23238_MOESM1_ESM.docx]

**Supplementary Information**

Supplementary Table S1. A previous study showed near-significant association at the CFA10 locus in a case-control GWAS within the American Cocker Spaniel (ACS) breed.^12^ Genotypes of the ACSs included in the current case-control GWAS at the CFA10 association are shown below (homozygous for the A allele [AA], heterozygous for the A and G alleles [AG], homozygous for the G allele [GG], or missing). The table shows the numbers and percentages of each genotype identified.

|  | Number (%) of dogs AA | Number (%) of dogs AG | Number (%) of dogs GG | Number (%) of dogs missing | Total |
| --- | --- | --- | --- | --- | --- |
| Cases | 24 (85.7) | 4 (14.3) | 0 (0.0) | 0 (0.0) | 28 |
| Controls | 15 (62.5) | 6 (25.0) | 3 (12.5) | 0 (0.0) | 24 |
